# Skeletal muscle growth dynamics and the influence of first-feeding diet in Atlantic cod larvae (*Gadus morhua* L*.*)

**DOI:** 10.1242/bio.018556

**Published:** 2016-09-09

**Authors:** Tu A. Vo, Trina F. Galloway, Tora Bardal, Christine K. Halseth, Gunvor Øie, Elin Kjørsvik

**Affiliations:** 1 Norwegian University of Science and Technology, Dept. of Biology, 7491 Trondheim, Norway; 2 SINTEF Fisheries and Aquaculture, P.O. box 4762 Sluppen, 7465 Trondheim, Norway

**Keywords:** Red and white muscle growth dynamics, Hyperplasia, Hypertrophy, Metamorphosis, First-feeding diet, Cod larvae, *Gadus morhua*

## Abstract

Dynamics between hypertrophy (increase in cell size) and hyperplasia (increase in cell numbers) of white and red muscle in relation to body size [standard length (SL)], and the influence of the first-feeding diets on muscle growth were investigated in Atlantic cod larvae (*Gadus morhua*). Cod larvae were fed copepod nauplii or rotifers of different nutritional qualities from 4 to 29 days post hatching (dph), *Artemia* nauplii from 20 to 40 dph and a formulated diet from 36 to 60 dph. The short period of feeding with cultivated copepod nauplii had a positive effect on both muscle hyperplasia and hypertrophy after the copepod/rotifer phase (19 dph), and a positive long term effect on muscle hypertrophy (60 dph). The different nutritional qualities of rotifers did not significantly affect muscle growth. We suggest here a model of the dynamics between hyperplasia and hypertrophy of red and white muscle fibre cells in relation to cod SL (4 to 30 mm), where the different red and white muscle growth phases clearly coincided with different metamorphosis stages in cod larvae. These shifts could be included as biomarkers for the different stages of development during metamorphosis. The main dietary muscle effect was that hypertrophic growth of red muscle fibres was stronger in cod larvae that were fed copepods than in larvae that were fed rotifers, both in relation to larval age and size. Red muscle fibres are directly involved in larval locomotory performance, but may also play an important role in the larval myogenesis. This can have a long term effect on growth potential and fish performance.

## Abstract (precis)

**Summary:** Hyperplastic and hypertrophic growth dynamics of red and white muscle were strongly related to cod larval size and corresponded with the metamorphosis process. First-feeding diet quality can prolong effects on muscle growth potential in cod larvae.

## INTRODUCTION

Fish muscle grows either by hyperplasia (recruitment of new muscle fibres) or by hypertrophy (increase in size of fibres already present) (Weatherley et al., 1988). In mammals and birds hyperplasia mostly stops after birth, whereas it continues for most of the adult life in teleost fish (Weatherley et al., 1988) and thus it is an important process in reaching the ultimate body size of large teleost fish species (Johnston, 1999). The dynamics between muscle hyperplasia and hypertrophy affect the somatic growth potential in teleost fish species (Weatherley and Gill, 1985; Weatherley et al., 1988). Muscle fibres are plastic in their growth response to environmental factors such as diet, exercise, light and temperature regimes (Johnston, 1999). Inadequate larval diets may lead to reduced muscle hyperplastic growth and cause a decreased somatic growth, even leading to muscle atrophy and late maturation (Leitão et al., 2011). Variation in nutritional conditions can produce different somatic growth rates in fish larvae, and the most rapidly growing larvae often have the highest rate of hyperplasia (Alami-Durante et al., 1997; Galloway et al., 1999; Ostaszewska et al., 2008; Leitão et al., 2011), which is thought to improve the growth potential in juvenile and adult stages (Galloway et al., 1999; Ostaszewska et al., 2008; Valente et al., 2013). Therefore, the study of muscle growth dynamics in the larval stages may be necessary to understand how environmental factors influence the growth potential in teleost fish.

Rotifers and *Artemia* sp. are commonly used as live food during first-feeding in intensive rearing systems for most marine fish larvae (Olsen, 2004). The use of harvested zooplankton as a first-feeding diet for marine fish larvae gives an improved survival, growth and development compared to the use of cultivated rotifers and *Artemia* sp. (Næss et al., 1995; McEvoy et al., 1998; Shields et al., 1999; Evjemo et al., 2003; Imsland et al., 2006; Rajkumar and Kumaraguru, 2006; Koedijk et al., 2010; Busch et al., 2010, 2011; Karlsen et al., 2015). First-feeding of harvested zooplankton to Atlantic cod larvae *Gadus morhua* L. has also led to a positive short- and long-term impact on growth (Imsland et al., 2006; Koedijk et al., 2010; Karlsen et al., 2015). Copepods are the natural zooplankton prey for fish larvae, and contain much higher levels of n-3 PUFAs (Evjemo et al., 2003), protein (Evjemo et al., 2003) and minerals (Mæhre et al., 2013) than cultivated rotifers and *Artemia* sp. However, several challenges relate to the use of wild zooplankton such as the limited harvesting season, sensitivity to handling and storage, and the potential transmission of parasites. The use of cultivated copepods is promising for improving the quality and quantity of marine juveniles, as was recently demonstrated for cod and ballan wrasse (*Labrus bergylta*) (Øie et al., 2015), where even short periods of cultivated copepods (*Acartia tonsa*) during first-feeding was beneficial.

Previous studies on the effect of early diets on muscle growth in fish larvae have mainly focused on white muscle (Alami-Durante et al., 1997; Galloway et al., 1999; Ostaszewska et al., 2008; Leitão et al., 2011). Although white muscle fibres constitute the main fillet yield and are active at high swimming speeds, red muscle fibres are active during slow sustained swimming movements (El-Fiky et al., 1987), and are hence of great importance for the functionality of the swimming musculature. Indeed, skeletal muscle is the largest and most rapid growing tissue in fish larvae (Osse et al., 1997; Johnston et al., 2011), but how the skeletal muscle growth dynamics correspond to the metamorphosis process has not yet been extensively studied.

The objectives of this study were therefore (a) to investigate the contribution of red and white muscle hyperplasia and hypertrophy to skeletal muscle growth in relation to cod larval body size (SL) and age (dph), and (b) to evaluate the effect of the first-feeding diet on red and white muscle growth in Atlantic cod larvae. The relationship between muscle growth dynamics and the process of metamorphosis in relation to SL in cod larvae was also discussed. This was done by analysing muscle development in cod larvae fed copepod nauplii or rotifers of different nutritional quality during the first 20 dph, before they all were weaned onto the same diets of *Artemia* nauplii followed by a formulated diet (Øie et al., 2015). One larval group was fed cultivated copepod nauplii to ensure optimal growth, another group was fed rotifers that were short-term enriched (the commercial hatchery standard) and the third group was fed rotifers that were not short-term enriched (‘poor nutritional standard’). The larvae were followed up to 60 dph. The characteristics of these larval diets and further details about the larval rearing, growth, survival and stress tolerance are described in Øie et al. (2015), where it was found that feeding with only copepods during the first 20 dph led to increased somatic growth compared to feeding with rotifers.

## RESULTS

### Growth under different live feed treatments

Cod larvae that were fed copepods were significantly bigger than larvae that were fed rotifers throughout the experiment. At 60 dph, the copepod-fed larvae were about twice as big as the rotifer-fed larvae, with a mean dry weight of about 22.3±1.4 mg per larva (Øie et al., 2015). The specific growth rate (SGR) of copepod-fed larvae was significantly higher (*P*<0.05) than that of RotMG- and RotChl-fed larvae in the copepod/rotifer phase (5-19 dph), in the weaning phase (33-60 dph), and for the whole experiment (5-60 dph), but not in the *Artemia* phase (19-33 dph) (Table 1). The highest SGR in all three treatments was in the *Artemia* phase (19-33 dph) (*P*<0.05; Table 1). The mean SL of the cod larvae was 4.6±0.1 mm on 4 dph, and at the end of the experiment (60 dph) the mean larval SL was significantly higher in the copepod treatment (25.7±1.9 mm) than in the rotifer treatments (*P*<0.05; Table 2). There were no differences in SGR or SL between RotMG and RotChl larvae (Tables 1 and 2).

**Table 1.**
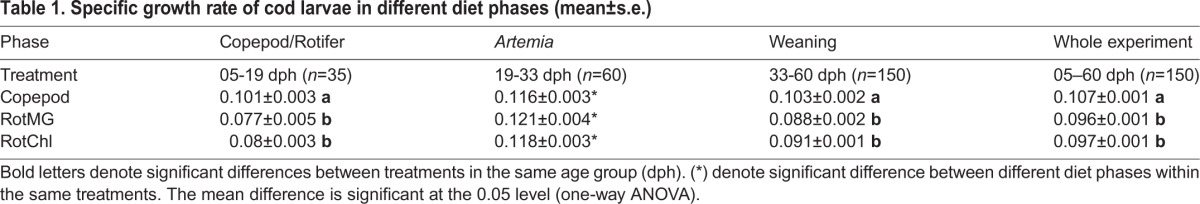
**Specific growth rate of cod larvae in different diet phases (mean±s.e.)**

**Table 2.**
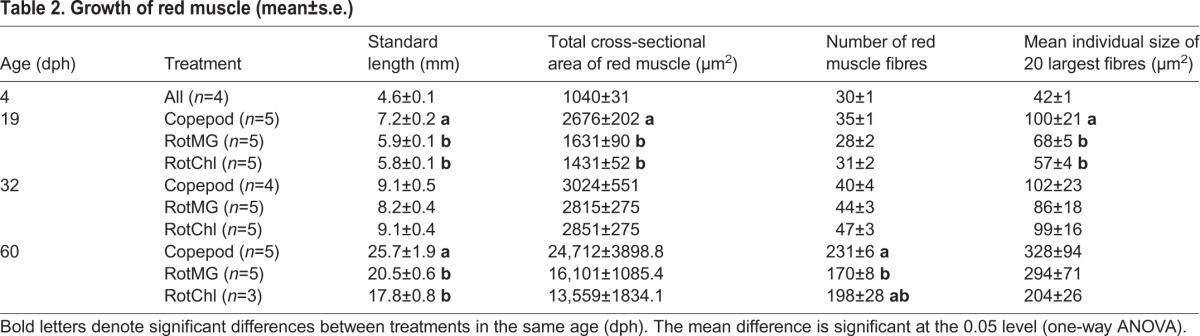
**Growth of red muscle (mean±s.e.)**

### Muscle fibre morphology

**Fig. 1.**
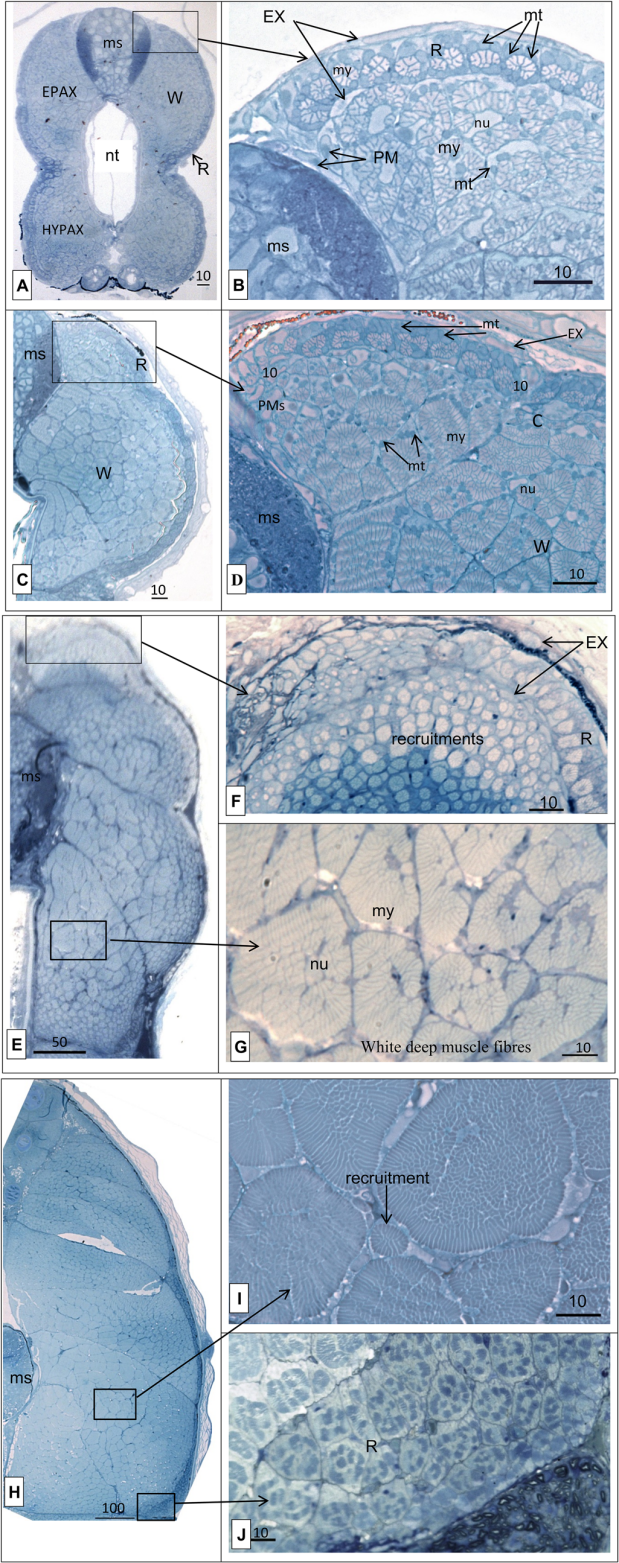
**Light micrographs showing transverse sections of skeletal muscle tissue immediately posterior to the anus in cod larvae.** (A) The shape of myotome at 4.6 mm SL (4 dph); (B) immature white and red muscle fibres and presumptive myoblasts, external cells at the apex of myotome at 4.6 mm SL; (C) the epaxial quadrant of the myotome at 6.3 mm SL (19 dph); (D) recruitment zone – stratified hyperplasia pattern and external cells at the apex of the myotome at 6.3 mm SL; (E) the epaxial quadrant of cod larva at 9.5 mm SL (32 dph); (F) recruitment zone at the apex of the myotome at 9.5 mm SL; (G) deep white muscle fibres at 9.5 mm SL; (H) the shape of an epaxial quadrant myotome of cod larva at 22 mm SL (60 dph); (J) several red muscle layers at the horizontal septum of cod larva at 22 mm SL; (I) a myotube forms on the surface of muscle fibres – mosaic hyperplasia pattern. ms, medulla spinalis; EPAX, epaxial quadrant of myotome; EX, external cell; HYPAX, hypaxial quadrant of myotome; mt, mitochondria; my, myofibrils; nt, notochord; nu, nucleus of myotube; PM, presumptive myoblast; R, red muscle fibres; SL, standard length; W, white muscle fibres; ms, medulla spinalis. Scale bars: 10 µm in A,B,C,D,F,G,I,J; 50 µm in E; 100 µm in H.

At a given SL, the muscle fibre morphology was similar in larvae from all treatments. At 4 and 19 dph (4.6-7 mm SL), the epaxial quadrant of myotome had a round shape and the deep white muscle fibres were covered by a single layer of red muscle fibres (Fig. 1A-D). Some presumptive myoblasts were observed at the apex of the myotome and between the red and white muscle fibre layers (germinal zones) (Fig. 1B and D). At 32 dph (8-10 mm SL) the epaxial quadrant myotomes became long and narrow in shape, and white muscle fibres were recruited intensively in germinal zones (Fig. 1E and F). The deep white muscle fibres present from hatching contained several bundles of myofibrils (Fig. 1B and D), which grew bigger and merged to one bundle of myofibril (Fig. 1G). The external cell layer was observed as a single layer of cells covering the outer red muscle layer, in 4-10 mm SL cod larvae (Fig. 1B,D and F). At 60 dph (16-31 mm SL), muscle fibres had grown strongly by hypertrophy, and the epaxial quadrant again had a rounder shape (Fig. 1H). A mosaic hyperplasia pattern of white muscle, in which white muscle recruitment fibres formed on the surface of existing muscle fibres, was only observed in cod larvae from around 22 mm SL (Fig. 1I). Several layers of red muscle fibres were observed in the 16-31 mm SL range (at 60 dph) (Fig. 1H and J).

### Muscle fibre growth in relation to larval age

The mean total cross-sectional area (CSA) of red muscle was significantly larger in copepod-fed larvae than in RotMG-fed larvae (*P*<0.001) and RotChl-fed larvae (*P*<0.001) after the copepod/rotifer phase (at 19 dph) (Table 2). At the same time, the hypertrophic growth of red muscle, as assessed by the mean size of the 20 largest red muscle fibres was significantly larger in copepod-fed larvae than in RotMG-fed larvae (*P*=0.01) and RotChl-fed larvae (*P*<0.001), but the mean number of red muscle fibres was not significantly different between the three treatments. After the *Artemia* phase (at 32 dph) there were no significant differences in red muscle fibre growth between the treatments. After the weaning phase (at 60 dph), the mean number of red muscle fibres was significantly higher in the copepod-fed larvae. The mean total CSA of red muscle and mean size of the 20 largest red muscle fibres were slightly larger in copepod-fed larvae than in the rotifer-fed larvae, although not significantly.

The mean total CSA of white muscle and the number of white muscle fibres were significantly larger in copepod-fed larvae than in in the rotifer-fed larvae (*P*<0.001) after the rotifer/copepod phase (at 19 dph) (Table 3). Also, the hypertrophic growth of white muscle fibres, as assessed by the mean size of the 50 largest white muscle fibres, was significantly larger in copepod-fed larvae than in RotMG-fed larvae (*P*=0.003) and RotChl-fed larvae (*P*<0.001). No significant differences in muscle growth between the three treatments were recorded after the *Artemia* phase (at 32 dph). After the weaning phase (at 60 dph), the mean total CSA of white muscle was significantly larger in copepod-fed larvae than in RotMG-fed and RotChl-fed larvae (*P*=0.035 and *P*=0.025, respectively). At this sampling point there was no significant difference in the mean number of white muscle fibres between the three treatments, but the mean size of the 50 largest white muscle fibres in copepod-fed larvae was again significantly larger than in both RotMG-fed larvae and RotChl-fed larvae (*P*=0.039 and *P*=0.037, respectively).

**Table 3.**
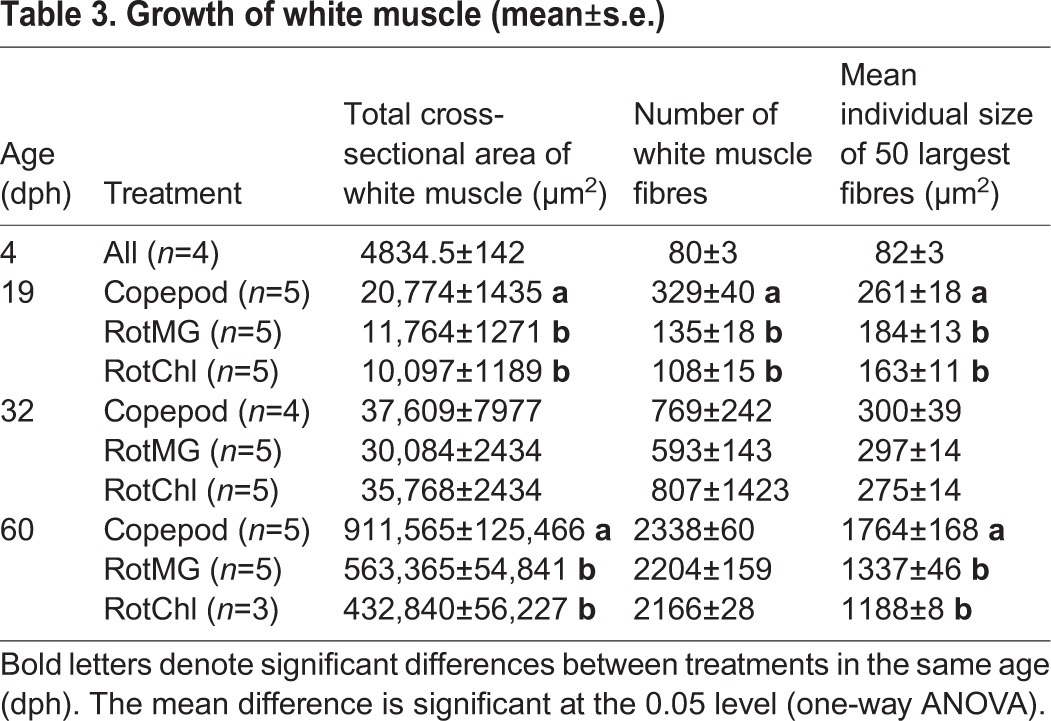
**Growth of white muscle (mean**±**s.e.)**

### Influences of first-feeding diet on muscle growth in relation to larval size

The number of red and white fibres, and the mean size of the 20 largest red muscle fibres and 50 largest white muscle fibres were strongly correlated with cod larval SL (Table 4). The 20 largest red muscle fibres at a given standard length was also significantly affected by the prey type (*P*<0.001). At 7.4 mm SL, the mean size of the 20 largest red muscle fibres was significantly larger in copepod-fed larvae (99.7±5 µm^2^) than in RotMG-fed larvae (82.5±3.8 µm^2^; *P*=0.001) and RotChl-fed larvae (75.2±3.7 µm^2^; *P*=0.001), estimated by a general factorial analysis of covariance (ANCOVA). No significant effect of prey type was found on white muscle growth related to larval size.

**Table 4.**
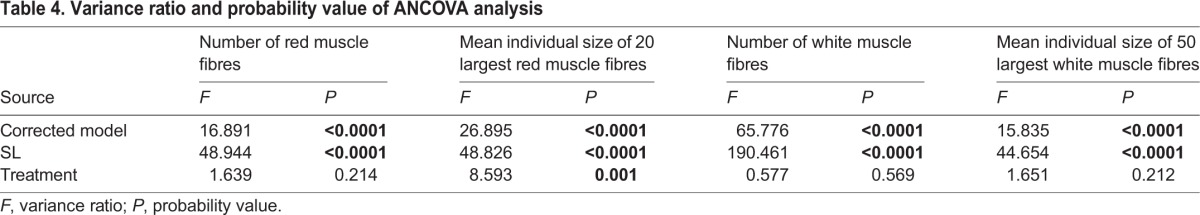
**Variance ratio and probability value of ANCOVA analysis**

### Muscle growth dynamics in relation to cod larval size

**Fig. 2.**
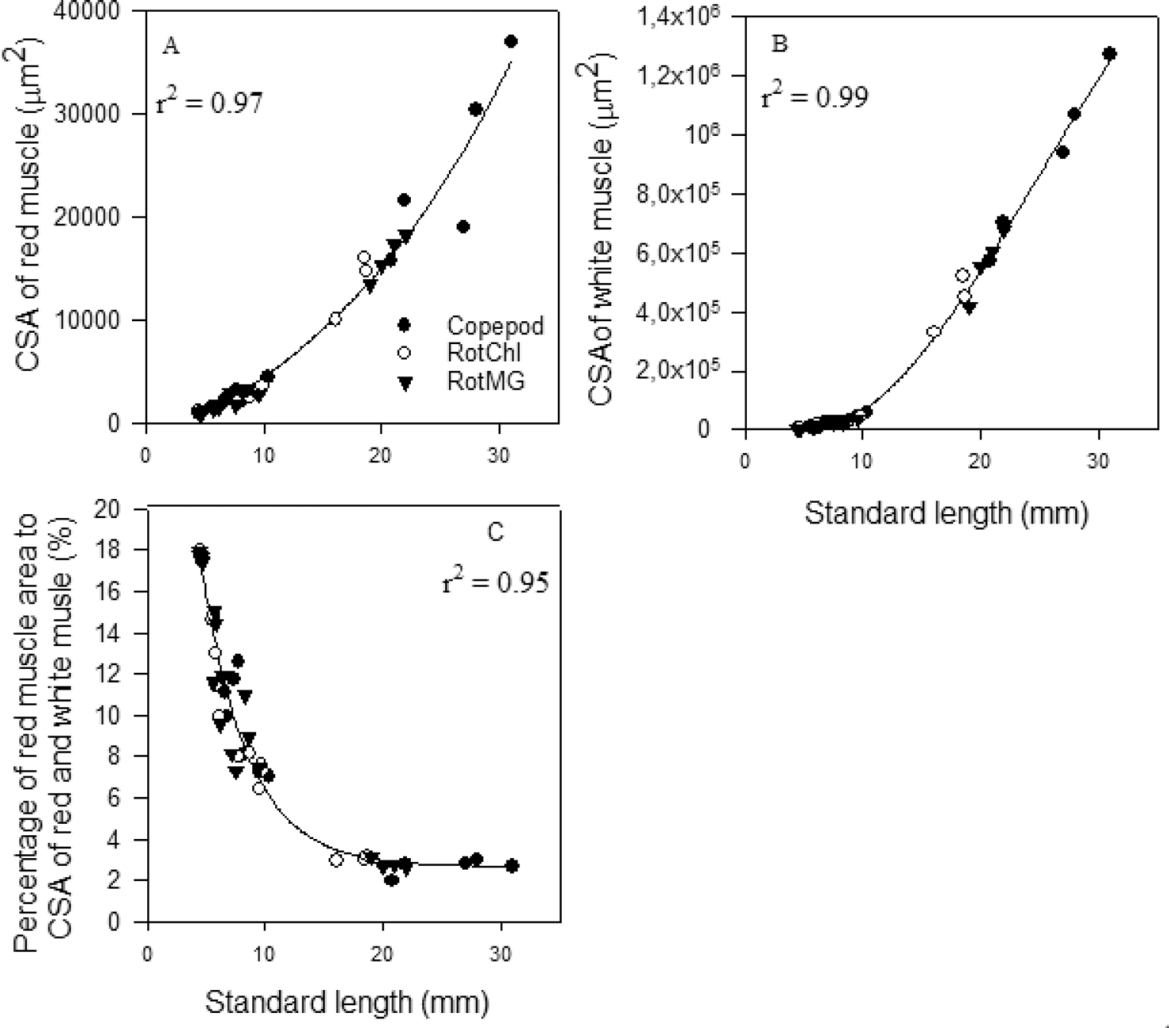
**The relationship between red and white muscle growth in one epaxial quadrant of the myotome and standard length (SL, mm) in cod larvae in three treatments (copepod-fed, RotMG-fed, and RotChl-fed).** (A) total cross-sectional area (CSA) of red muscle (µm^2^), (B) total CSA of white muscle (µm^2^), and (C) percentage of red muscle area to total red and white muscle area (%); each point represents one cod larva. Regression line equations are as follows: (A) y=9484×1.05^X^−11,315 (r^2^=0.97); (B) y=−59x^3^+4452x^2^−44,486x+124,754 (r^2^=0.997; *P*<0.0001); (C) y=47exp(−0.25x)+2.6 (r^2^=0.95; *P*<0.0001). Nonlinear regression - dynamic curve fitting was performed for all cod larvae.

The mean CSA of red muscle had an exponential growth (Fig. 2A, r^2^=0.97, *P*<0.001), and the white muscle had a polynomial growth (Fig. 2B, r^2^=0.99, *P*<0.001), when correlated with SL. The percentage of CSA of red muscle to CSA of total muscle had an exponential decline with increasing SL (r^2^=0.95, *P*<0.001); it decreased sharply from 18 to 6% when SL increased from 4 to 10 mm, after which it decreased slowly from 6 to 3% when SL increased from 10 to 20 mm, and stabilised around 3% in the size range 20-30 mm SL (Fig. 2C).

**Fig. 3.**
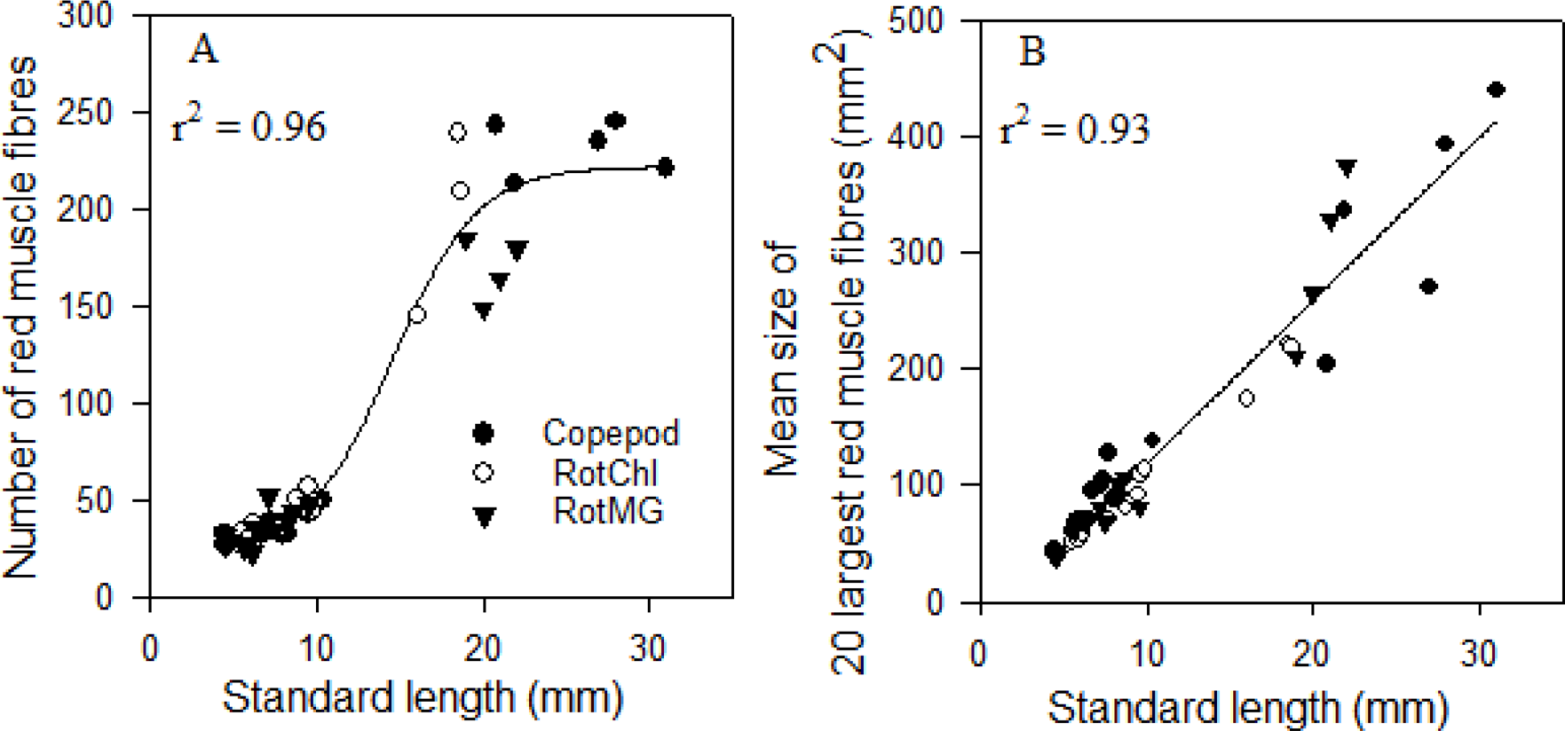
**The red muscle growth dynamics in relation to standard length (SL, mm) in cod larvae in three treatments (copepod-fed, RotMG-fed, and RotChl-fed).** (A) The number of red muscle fibres, (B) the mean size of 20 largest red muscle fibres. Each point represents one cod larva. Regression line equations are as follows: (A) y=197/(1+exp (−(x-15)/2.5))+25 (r^2^=0.95; *P*<0.0001); (B) y=14x−21 (r^2^=0.93; *P*<0.0001). Nonlinear regression - dynamic curve fitting was performed for all cod larvae.

The number of red muscle fibres had a sigmoidal growth correlation with larval SL (Fig. 3A, r^2^=0.96, *P*<0.0001) with three different phases. The first phase was a lag phase (number of muscle fibres increased slowly) while the larvae grew from 4.6 to 10 mm SL, the second phase was a log phase (number of muscle fibres increased exponentially) when the larval SL increased from 10 to 20 mm, and the third phase was a stationary phase (number of muscle fibres stabilised) while the larvae grew from 20 to 30 mm SL. The mean individual size of the 20 largest red muscle fibres had a positive linear correlation with SL (Fig. 3B, r^2^=0.93, *P*<0.0001).

**Fig. 4.**
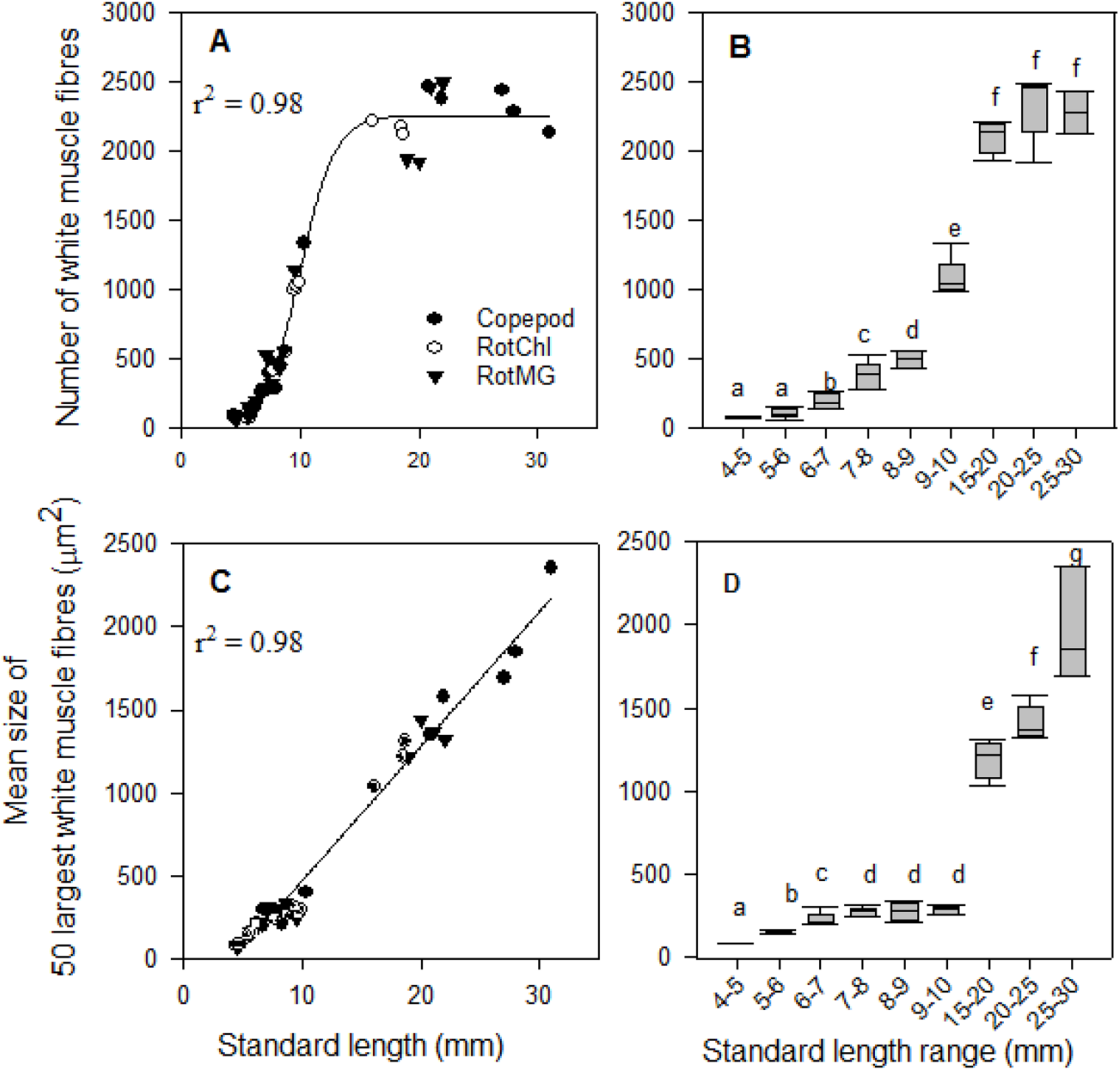
**The white muscle growth dynamics in relation to standard length (SL, mm) in cod larvae in three treatments (copepod-fed, RotMG-fed, and RotChl-fed).** (A) The number of white muscle fibres, each point represents one cod larva; (B) the mean number of white muscle fibres in different SL ranges (*n*=4, 7, 5, 7, 4, 6, 4, 5, 3); (C) the mean size of the 50 largest white muscle fibres; (D) the mean size of 50 largest white muscle fibre in different SL ranges (*n*=4, 7, 5, 7, 4, 6, 4, 5, 3), each point represents one cod larva. Regression line equations are as follows: (A) y=2257/(1+exp (−(x−10)/1.5)) (r^2^=0.98; *P*<0.0001); and (C) y=81x−336 (r^2^=0.98; *P*<0.0001); small letters (a-f in B, a-g in D) denote significant differences between different SL ranges (*P*<0.05). A one-way ANOVA was performed to determine significant differences between size groups.

The number of white muscle fibres also had a sigmoidal correlation with larval SL (Fig. 4A, r^2^=0.98, *P*<0.001); in the first lag phase, the mean number of white muscle fibres did not increase significantly while the larval SL increased from 4 to 6 mm (Fig. 4B), in the second log phase, the mean number of white muscle fibres increased exponentially while the cod larval SL increased from 6 to 15 mm (Fig. 4A, *P*<0.05), and in the stationary phase, the mean number of white muscle fibres did not increase significantly while the larval size increased from 15 to 30 mm (Fig. 4B). The mean individual size of the 50 largest white muscle fibres had a linear correlation with larval SL (Fig. 4C, r^2^=0.98, *P*<0.001), but did not increase significantly while the cod larval SL increased from 7 to10 mm SL (Fig. 4D).

**Fig. 5.**
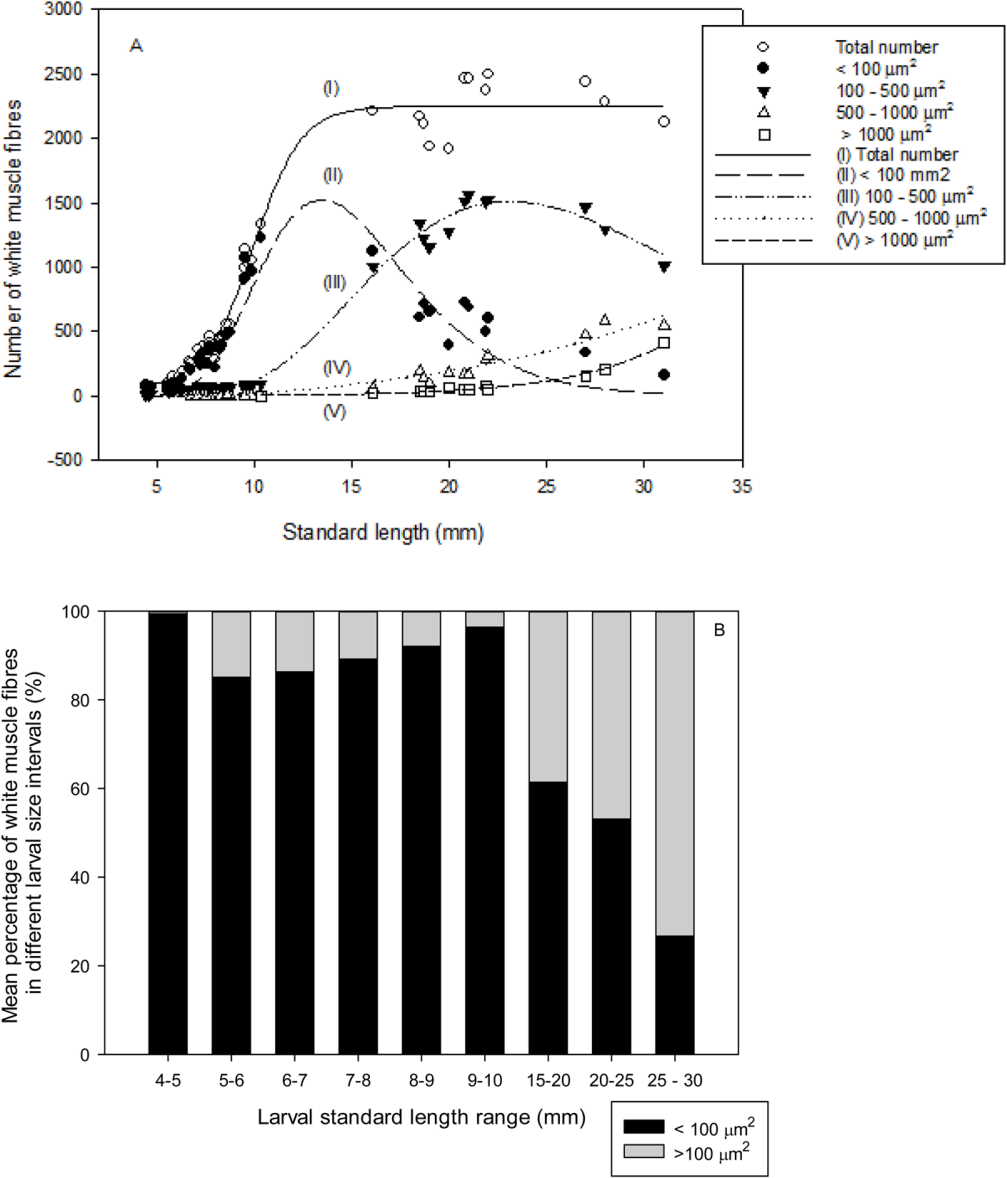
**The model of white muscle growth dynamics between hyperplasia and hypertrophy in cod larvae in three treatments (copepod-fed, RotMG-fed, and RotChl-fed l) in relation to standard length (SL, mm).** (A) The number of white muscle fibres within each size interval. Each point represents one cod larva. I, the total number of white muscle fibres; II, the number of white muscle fibres <100 µm^2^; III, the number of white muscle fibres 100-500 µm^2^; IV, the number of white muscle fibres 501-1000 µm^2^; V, the number of white muscle fibres >1000 µm^2^. Regression line equations are as follows: I, y=2195/(1+exp (−(x−10)/1.3))+58 (r^2^=0.98; *P*<0.0001); II, y=21,317×exp(−0.5×(ln(x/14.5)/0.28)^2^)/x (r^2^=0.96; *P*<0.0001); III, y=37,280×exp (−0.5(ln(x/26)/0.37)^2^)/x) (r^2^=0.99; *P*<0.0001); IV, y=51×1.1^x^−94 (r^2^=0.94; *P*<0.0001); V, y=0.62×1.2^x^ (r^2^=0.99; *P*<0.0001). Nonlinear regression - dynamic curve fitting was performed for all cod larvae to study the relationship between muscle growth and standard length. (B) Mean percentage of white muscle fibres smaller and larger than 100 µm^2^ in one epaxial quadrant of the myotome in relation to the different cod larval size intervals (*n*=4, 7, 5, 7, 4, 6, 4, 5, 3; same SL range as in Fig. 4B and D).

White muscle growth dynamics between hyperplasia and hypertrophy in relation to larval SL, was studied by a model based on number of white muscle fibres within the different cod larval size intervals (Fig. 5). The number of the smallest white muscle fibres (<100 µm^2^) was stable from 4 to 6 mm SL, increased exponentially from 6 to 12 mm SL, peaked between 12 and 15 mm, and then decreased sharply from 15 to 30 mm SL (Fig. 5A). The number of medium sized white muscle fibres (101-500 µm^2^) was stable from 4 to 10 mm SL, increased exponentially from 10 to 20 mm SL, peaked between 20 and 25 mm, and then decreased slowly from 25 to 30 mm SL. The number of larger white muscle fibres (500-1000 µm^2^ and >1000 µm^2^) increased slowly from 10 to 20 mm SL, and exponentially from 20 to 30 mm SL (Fig. 5A). The percentage of the smallest white muscle fibres (<100 µm^2^) decreased from around 99% to around 80% while the cod larval SL increased from 4 to 6 mm, then increased again up to about 95% when the cod larval SL reached about 10 mm, and then decreased continuously to around 35% when the cod larval SL was around 25 mm (Fig. 5B).

## DISCUSSION

### Muscle growth dynamics

**Fig. 6.**
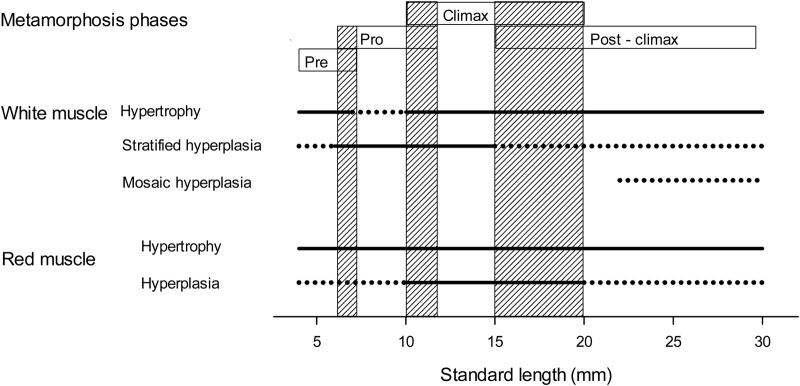
**Muscle growth dynamics in relation to standard length (SL, mm) and stage of metamorphosis in cod larvae.** Skeletal muscle growth of both red and white muscle by both hypertrophy and hyperplasia mechanisms. Solid line, predominant muscle growth mechanism; broken line, non-dominant muscle growth mechanism; shaded boxes, transition between different metamorphosis phases.

The present study demonstrated that muscle growth and the dynamics between hyperplasia and hypertrophy were positively correlated to cod larval size (SL), rather than to larval age. To our knowledge, we here suggest the first model of the dynamics between hyperplasia and hypertrophy of white muscle fibres related to cod larval size (SL) (Fig. 5). Our results showed that muscle growth dynamics in cod larval red and white muscles can be divided into different phases that correspond with different stages in the process of cod metamorphosis, as summarized in Fig. 6. In the larval size range that we studied, the white muscle growth dynamics were divided into at least four main phases. The white muscle grew mainly by hypertrophy while the cod larval SL increased from 4–7 mm SL, and by hyperplasia while cod larval SL increased from 6 to12 mm SL (especially from 7 to10 mm SL); hypertrophy and hyperplasia seemed equal while cod larval SL increased from 10 to 15 mm SL, and hypertrophy dominated thereafter. The red muscle growth dynamics was divided into three main growth phases (4 to 10; 10 to 20; and 20 to 30 mm SL). Red muscle grew by hypertrophy during the whole period, whereas red muscle hyperplasia was exponential while the cod larval SL increased from 10 to 20 mm. These changes in muscle growth phases also corresponded with different stages of the metamorphosis in cod larvae.

Towards the end of the copepod/rotifer feeding (around 6 to 7 mm SL, about 19 dph), the white muscle growth in cod larvae changed from the initial hypertrophy to the second growth phase characterized by stratified hyperplasia, leading to a more oblong and narrow shape of the myotome. The 6–12 mm size range represents a period of very rapid change in cod larval organ development, where from 6 mm SL the gut is forming a loop and enterocytes go through a very rapid hypertrophy (Kjørsvik et al., 1991; Wold et al., 2008). At around 20 dph, there is also a shift in the major digestive enzymes gene expression patterns (Kortner et al., 2011). Onset of ossification of the spinal column in cod is observed from 6 to 7 mm SL, and all vertebral elements are ossified by 11-12 mm SL (Kjørsvik et al., 2009). The formation of fin rays in dorsal and ventral unpaired fins have been used as landmark indicators for the onset of metamorphosis, demonstrating that pro-metamorphosis starts around 6 mm SL in cod larvae (von Herbing Hunt et al., 1996). These observations suggest that the first change in muscle growth around 6-7 mm SL coincides with the transition of cod larvae from pre-metamorphosis to the pro-metamorphosis stage. Hamre et al. (2014) found a shift in the redox regulation of cod larvae around 20 dph (<7 mm SL), and hypothesised that the start of mosaic hyperplasia could be the main reason for this ontogenetic shift. We did not find mosaic hyperplasia in cod larval white muscle until 22 mm SL in the present study, and we suggest that the ontogenetic change in redox regulation is rather following the shift into the pro-metamorphic stage, where several major changes occur in vital organs.

We found that the white muscle of cod larvae entered into a third growth phase around 10-12 mm SL, from growing mainly by hyperplasia to equally by hypertrophy and hyperplasia. Also at this point the red muscle growth changed from mainly by hypertrophy to by both hypertrophy and hyperplasia. Other characteristic changes are found in cod larvae around 10 mm SL, when the midgut epithelium volume, the number of enterocytes, and the total microvilli surface area changes from gradual growth to exponential growth, and the gut mitochondria go through a maturation phase (Wold et al., 2008). The appearance of a functional stomach and pyloric caeca is often used as an indicator of the climax-metamorphosis in teleost larvae (Tanaka, 1971; Govoni et al., 1986), and the formation of these have been observed in cod larvae from around 10-12 mm SL (Pedersen and Falk-Petersen, 1992; Perez-Casanova et al., 2006). The larval median finfold is resorbed during climax-metamorphosis in teleost larvae (Balon, 1975), and this occurs in cod larvae between 11 and 15 mm SL (Kvenseth and Øiestad, 1984; Pedersen and Falk-Petersen, 1992). The results suggest that the size range between 10 and 12 mm SL, when cod larvae turn from pro-metamorphosis to climax-metamorphosis, corresponds to the observed changes in the white and red muscle.

Around 15 mm SL, the white muscle entered into the fourth growth phase, where hypertrophy dominated, and the myotome shape became rounder again. Around 20 mm SL, the red muscle growth changed to mainly hypertrophy. Hypertrophy is the dominant mechanism of muscle growth in juvenile and adult fish (Rowlerson et al., 1995; Patruno et al., 1998). Compared with some other teleost species, the transition from larva to juvenile appears prolonged in Atlantic cod. The gastric glands in the stomach can be observed during climax-metamorphosis (see above). Pepsinogen gene expression was first found in cod larvae around 60 dph (Perez-Casanova et al., 2006; probably corresponding to 18-20 mm SL), and a fully developed stomach and pyloric caeca was found from 45 mm SL (Pedersen and Falk-Petersen, 1992). In several cod larval rearing experiments, cod larvae have coped well with formulated juvenile diets from around 20 mm SL (Hamre et al., 2014; Karlsen et al., 2015). Therefore, the more mature digestive system of cod larvae should function from around 20 mm SL, although the ratio of pyloric caeca length to fish SL still increases with increasing SL from 20 to 45 mm SL (Pedersen and Falk-Petersen, 1992). We observed the onset of mosaic hyperplasia growth in white muscle from around 22 mm SL. This is an important growth pattern in juveniles and adults of teleost fish that reach a large final size (Greer-Walker, 1970; Weatherley et al., 1988; Johnston, 1999). We suggest that the changes in predominant growth patterns in white and red muscle for cod larvae at 15-20 mm SL are associated with the transition from climax-metamorphosis to post-climax-metamorphosis.

We found that the dynamics of red and white muscle growth clearly coincided with different metamorphosis stages in cod larvae (Fig. 6). The stratified hyperplastic growth started around 5.5 mm SL and was intense from around 6 mm SL, (as also described by Galloway et al., 1999), and the mosaic hyperplasia pattern was observed from about 22 mm SL. We suggest that the onset of stratified hyperplasia and mosaic hyperplasia in white muscle growth can be used as a muscle indicator for the onset of metamorphosis and post-climax-metamorphosis, respectively, in cod larvae.

### The influence of first-feeding diet

To our knowledge, this is the first time a significant positive and long-term effect from diet quality is observed on red muscle development. The red muscle fibres were larger when cod larvae were fed copepods rather than rotifers at a given SL (5–10 mm SL range). The contribution of red muscle to skeletal muscle was highest (around 18% total CSA) in the copepod/rotifer phase, and less (about 3% total CSA) in the weaning phase. The influence on red muscle growth in small larvae will thus have especially strong effects on larval locomotory performance, which may impact larval growth and survival. The copepod-fed larvae from the present experiment indeed had a different activity pattern, were more effective predators, and had less vertebral anomalies than the rotifer- fed larvae (E.K., T.B., Marit Hansen, G.Ø., unpublished data). They were also more tolerant to handing stress than those fed rotifers (Øie et al., 2015).

Furthermore, muscle specification is under the control of complex signalling pathways from the surrounding tissues (Johnston et al., 2011). In early segmentation-stage embryos in teleost fish, three distinct myogenic compartments, adaxial, anterior and posterior cells are spatially segregated within newly formed somites (Hollway et al., 2007). The adaxial cells, found next to the notochord, requires signalling from hedgehog (Hh) proteins, secreted from the notochord to specify formation of the red muscle cells (Wolff et al., 2003; Hirsinger et al., 2004). Specification of posterior cells to form the white muscle cells are triggered by the migration of adaxial cells (Henry and Amacher, 2004) and formation of the red muscle fibres. The anterior cells become positioned externally to the red muscle, and become the external cell layer, after the process of somite rotation during mid-segmentation (Hollway et al., 2007). External cells are a source of muscle precursor cells that may provide a second wave of myogenesis, as a stratified hyperplasic growth in late embryonic or in early larval stages in teleosts (Hollway et al., 2007). The external cells may also provide a population of quiescent muscle precursor cells for later stages (Hollway et al., 2007; Stellabotte et al., 2007). The signalling requirements for specification of external cells to form stratified hyperplasia growth pattern is not known. However, the red muscle fibres are the closest tissue of external cells, so they could play an important role in the second wave of myogenesis process in teleosts. Thus, the role of red muscle in the myogenesis process of teleost fish needs further study.

External cells surrounding the red muscle layer were observed in 4–10 mm SL cod larvae, and the stratified hyperplasia growth pattern occurred during the whole live-prey feeding phase of cod larvae (4–15 mm SL). The live-prey feeding period is known as the most challenging in marine aquaculture species, due to high mortalities and incidences of deformities. However, there has not been much emphasis on possible effects on the muscle growth potential, one of the most important features in aquaculture species. In a later experiment, cod larvae were fed natural zooplankton or rotifers/*Artemia* during the whole first-feeding period, and they were followed up to 2 years old. At a given SL, there was also no effect of the first-feeding diet on the stratified hyperplasia, but we found a long-lasting effect on the mosaic hyperplasia in white and red muscle (T.A.V., T.F.G., T.B., Ivar Rønnestad, Kristin Hamre, Terje van der Mehren, Ørjan Karlsen, E.K., unpublished data). Therefore, the first-feeding nutritional quality is not only important for the survival of cod larvae, but may also play an important role in their potential growth in later stages. Indeed, the myogenesis process, beginning with the stratified hyperplasia pattern and then later with the mosaic hyperplasia pattern, has also been observed in many pelagic larvae such as sea bass (*Dicentrarchus labrax*) (Veggetti et al., 1990), sea bream (*Sparus aurata*) (Rowlerson et al., 1995), Atlantic herring (*Clupea harengus*) (Johnston et al., 1998), and pike perch (*Sander lucioperca*) (Ostaszewska et al., 2008). Thus, the hyperplasia growth phase can be an open window for modulating growth potential by early nutritional quality in pelagic larval species.

Larval dietary quality has affected white muscle hyperplasia in larvae of common carp (Alami-Durante et al., 1997), Atlantic cod (Galloway et al., 1999), pike perch (Ostaszewska et al., 2008), and pacu (Leitão et al., 2011). However, in these experiments the larvae were followed in only short periods and ended when the larvae were still receiving the different diets. In the present study, we were able to demonstrate that a short period of feeding with cultivated copepod nauplii had a positive effect on both muscle hyperplasia and hypertrophy in the copepod/rotifer phase, and we observed a positive long-term effect on muscle hypertrophy in the weaning phase. In this study, we demonstrated that using cultivated copepods for the first 2-3 weeks of first-feeding had a long lasting and positive effect on growth and survival of the cod larva until the end of the experiment at 60 dph. This was similar to the improved growth rate in cod larvae when using natural zooplankton dominated by copepods during the whole larval period (Imsland et al., 2006; Koedijk et al., 2010; Karlsen et al., 2015).

### Conclusions

We found a strong correlation between muscle growth patterns and cod larval size. Changes in the dynamics between hyperplasia and hypertrophy in red and white muscle coincided with the different metamorphosis stages in cod larvae, and these shifts in muscle growth dynamics could be included as biomarkers for the different stages of development during metamorphosis. The onset of stratified hyperplasia and mosaic hyperplasia in white muscle were associated with the onset of the pro-metamorphosis and post-climax-metamorphosis stages, respectively. The hypertrophic growth of red muscle fibres was stronger in cod larvae that were fed copepods than in larvae that were fed rotifers, both in relation to larval age and to larval size. Red muscle fibres are not only involved in the larval locomotory performance, but may also play an important role in the larval myogenesis process. This can have a long-term effect on growth potential and fish performance. The early muscle growth is therefore a critical indicator in nutritional studies of marine fish species.

## MATERIAL AND METHODS

### The experiment

The experiment was performed according to the national regulations related to laboratory animal welfare, and all researchers and technical staff involved were certified according to the requirements for Felasa C, approved by The Norwegian Animal Research Authority (NARA).

Atlantic cod (*Gadus morhua*) eggs were obtained 2 days before hatching from a broodstock spawning tank at Nofima's Cod Breeding Center (Tromsø, Norway), and transferred to 100-l cone-bottomed tanks with seawater (34 ppt) with a density of 100 eggs l^−1^. The water temperature in the rearing tanks was 6°C until hatching and increased gradually to 12°C during the first 15 days after hatching.

Three different first-feeding treatments were established, where cod larvae were fed either copepod nauplii or rotifers of two different nutritional qualities during the earliest first-feeding period. Each treatment had three replicate tanks. To ensure equal prey density and prey size for all larvae, copepod nauplii (*Acartia tonsa*) (stage NIII-V, 170-210 µm) and rotifers (*Branchionus ibericus*) (180 µm in adult lorica length) of equivalent sizes (Alves et al., 1999; Penglase et al., 2010) were used for rearing the cod larvae from 3 to 28 dph, and the cod larvae were fed three times per day at a prey density of 12,000 litre^−1^. In the copepod treatment, cod larvae (copepod-fed larvae) were fed with *A. tonsa* nauplii, cultivated on the micro algae *Rhodomonas baltica*. In the RotMG treatment, cod larvae (RotMG-fed larvae) were fed with rotifers (*B. ibericus*), cultivated on the algal paste DHA *Chlorella* (Chlorella industry co. Ltd, Japan) and short-term enriched with Multigain (BioMar AS, Norway). In the RotChl treatment, cod larvae (RotChl-fed larvae) were fed rotifers (*B. ibericus*) cultivated on the algal paste DHA *Chlorella*, but without any short-term enrichment. All larval groups were fed *Artemia* sp. nauplii enriched with Multigain (Biomar AS, Norway) from 20 to 40 dph, and a formulated diet (Gemma micro 300, Skretting, Norway) from 36 to 60 dph (10 g dry feed per tank day^−1^). The details of larval rearing conditions and cultivation and analyses of the first feeding organisms are described in Øie et al. (2015).

### Growth and survival

The larval dry weight and survival are described in Øie et al. (2015) and was followed during the whole experiment (2-60 dph). The specific growth rates (SGR) of cod larvae was calculated according to Ricker (1958):

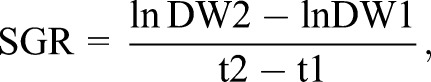

where DW1 is the mean larval dry weight at sampling time t1, and DW2 is the larval dry weight at sampling time t2.

### Larval sampling and muscle analysis processing

The larvae were collected randomly from all experimental tanks for muscle analysis. The sampled larvae were then anaesthetized in Tricaine Methanesulfonate (MS222, Finquel, Argent Chemical Laboratories Inc., Redmond, USA) before further treatment. Five larvae were sampled before feeding (4 dph), and 5 larvae from each tank were sampled at the end of the copepod/rotifer phase (19 dph), at the end of the *Artemia* phase (32 dph), and 20 days after weaning onto the formulated diet (60 dph) (Table 1). The fish larval samples were fixed in 2.5% paraformaldehyde and 2.5% glutardialdehyde in 0.08 M cacodylic buffer (Galloway et al., 1998). All samples were stored at 4°C until further processing. SL was measured from the tip of the snout to the end of the notochord before embedding in Epon. Transverse semi-thin (1 µm) sections, made on a Leica Reichert Ultracut microtome (Leica Microsystems, Germany) were cut just behind the anus. The sections were stained in Nile blue (McGree-Russell and Smale, 1963) for 2 h at 60°C. The sections were photographed using a Zeiss Axioskop 2 plus microscope (Zeiss INC., Germany), equipped with a Nikon DS-5 M camera (Nikon corp., Japan). Photographs of the sections were processed in the open software ImageJ (Wayne Rasband, NIH, Bethesda, MD, USA) in a LCD table drawing board, DTK-2400 (Wacom, China). All red and white muscle fibre cells in one epaxial quadrant of the cod larval myotome were measured through the photographs. The external cell layer was identified as described by Rowlerson et al. (1995).

### Calculation and estimation of muscle growth

The cross sectional area (CSA) of the red and white muscle was calculated as the sum of all red and white muscle cell areas in one epaxial quadrant of the cod larval myotome, which was used to calculate the contribution of red and white muscle fibres to growth in relation to cod larval age (dph) and size (SL).

White and red muscle hyperplasia were assessed by the numbers of white and red muscle fibres per epaxial quadrant of the cod larval myotome. White and red muscle hypertrophy were assessed by the mean size of the 50 largest white muscles fibres and the 20 largest red muscle fibres, respectively, and which represented the number of fibres present of one epaxial quadrant of the cod larval myotome at hatching (Galloway et al., 1998).

A model based on the number of white muscle fibres within each size interval (<100, 101-500, 501-1000, >1000 µm^2^) in relation to larval SL was used to evaluate the contribution of white muscle hypertrophy and hyperplasia at a given SL. Recruitment fibres have often been defined as the fibre diameter smaller than 10 or 20 µm in teleost fish (Weatherley and Gill, 1985; Koumans et al., 1993; Johnston et al., 1998; Ostaszewska et al., 2008). Therefore, white muscle hyperplasia was assessed by the number of the smallest white muscle fibres (<100 µm^2^) and white muscle hypertrophy was assessed by the number of white muscle fibres in the larger size ranges (101-500, 501-1000, >1000 µm^2^). Fish muscle grows by both hyperplasia and hypertrophy (Weatherley et al., 1988); thus, at a given SL, the contribution of white muscle hypertrophy and hyperplasia was defined as below.

Predominantly hyperplasia: if the total number of white muscle fibres and the number of the smallest white muscle fibres increased, the number of new muscle fibres generated was higher than the number of muscle fibres getting larger in size.

Equal hyperplasia and hypertrophy: if the total number of white muscle fibres increased and the number of the smallest white muscle fibres was stabilised (peak), the number of new muscle fibres generated was equal to the number of muscle fibres getting larger in size.

Predominantly hypertrophy: if the total number of white muscle fibres was stable and the number of the smallest white muscle fibres decreased, white muscle fibres were mainly growing to a larger size.

If the number of white muscle fibres in the larger size ranges (101-500, 501-1000, >1000 µm^2^) increased, hypertrophy was ongoing.

### Statistical analysis

Results were given as mean values±standard error. The software SigmaPlot12.3 for Windows was used for the statistical analyses. Normality in the data was tested using a Shapiro-Wilk's test. Different means were compared by using a one-way ANOVA (analysis of variance) with Student–Newman–Keuls *post hoc* test for homogenous data, and with Dunnett T3 *post hoc* test for non-homogenous data. For non-normal distributed data, the non-parametric Kruskal–Wallis was applied. A value of *P*<0.05 was considered significant.

The relationship between muscle growth and SL was tested by using curve fitting for all cod larvae in the three treatments. From 5.5-10 mm SL (on 19 and 32 dph), a linear regression relationship was found between the number of red and white muscle fibres and SL. Therefore, only cod larvae from 5.5-10 mm SL (19 and 32 dph cod samples) were used for studying the direct influences of the first-feeding treatment on red and white muscle growth in relation to SL, by using a general factorial analysis of covariance (ANCOVA) with treatment type as a fixed factor and SL as a covariate in the program IBM SPSS statistic 20 for Windows.
